# Kynurenic Acid/GPR35 Signaling Protects the Infarcted Heart by Suppressing Macrophage mtDNA-Triggered cGAS-STING Activation

**DOI:** 10.3390/antiox15030300

**Published:** 2026-02-27

**Authors:** Yuyuan Mao, Jiao Jiao, Xinyu Zhu, Wenhu Liu, Shujie He, Nana Li, Haoyi Yang, Jingyong Li, Tingting Tang, Ni Xia, Xiang Cheng

**Affiliations:** 1 Department of Cardiology, Union Hospital, Tongji Medical College, Huazhong University of Science and Technology, Wuhan 430022, China; elonyuyuan@163.com (Y.M.); jjpatricia@163.com (J.J.);; 2 Hubei Key Laboratory of Biological Targeted Therapy, Union Hospital, Tongji Medical College, Huazhong University of Science and Technology, Wuhan 430022, China; 3 Hubei Engineering Research Center for Immunological Diagnosis and Therapy of Cardiovascular Diseases, Union Hospital, Tongji Medical College, Huazhong University of Science and Technology, Wuhan 430022, China

**Keywords:** myocardial infarction, macrophage, kynurenic acid, GPR35, cGAS, STING

## Abstract

Kynurenic acid (KynA), a tryptophan metabolite that regulates immune homeostasis via G protein-coupled receptor 35 (GPR35), has an undefined role in post-myocardial infarction (MI) immune responses. To clarify this role, we established a murine MI model and administered KynA intraperitoneally to evaluate cardiac function and ventricular remodeling. Macrophage infiltration was assessed, and macrophages were depleted via clodronate liposomes to confirm their contribution to KynA-mediated cardioprotection. In bone marrow-derived macrophages (BMDMs), GPR35-targeted siRNA verified the receptor-dependent action of KynA. KynA improved cardiac function, reduced infarct scarring and fibrosis, and suppressed pro-inflammatory macrophage infiltration in MI mice, with these cardioprotective effects abrogated by macrophage depletion. Mechanistically, KynA inhibited voltage-dependent anion channel 1 oligomerization, prevented mitochondrial DNA leakage, and downregulated the cGAS/STING/TBK1/IκBα/P65 pathway in macrophages, while exogenous mitochondrial DNA counteracted this inhibition. Collectively, the KynA/GPR35 axis exerts cardioprotective effects against MI by attenuating macrophage pro-inflammatory responses, highlighting its potential as a novel therapeutic target.

## 1. Introduction

Myocardial infarction (MI) persists as one of the top contributors to global morbidity and mortality despite significant advances in reperfusion and antithrombotic therapies [1]. Beyond the initial ischemic insult, the subsequent sterile inflammatory response critically influences infarct healing, ventricular remodeling, and long-term cardiac function [2,3]. The appropriate modulation of these inflammatory processes remains a central challenge in cardiovascular medicine.

Kynurenic acid (KynA), a neuroactive metabolite derived from tryptophan catabolism via the kynurenine pathway, has been widely investigated in the central nervous system [4,5]. KynA reduces ischemia-induced brain injury [6] and neuronal cell death [7]. KynA has recently emerged as an important immunoregulatory molecule. It suppresses pro-inflammatory cytokine production [8,9], modulates T cell differentiation [10], and inhibits inflammasome activation [9]. However, other studies have revealed that KynA may enhance leukocyte adhesion and tissue infiltration [11,12], emphasizing that its biological effects are strongly context-dependent.

G protein-coupled receptor 35 (GPR35) has been identified as a functional receptor for KynA and other endogenous ligands [13], and accumulating evidence has implicated it in leukocyte trafficking and cytokine regulation [14,15,16,17,18]. In cardiovascular research, activation of GPR35 has been linked to cardioprotection in ischemia–reperfusion models [19].

Macrophages, critical constituents of the immune system, play a pivotal role in cardiac repair after myocardial infarction. These cells exert context-dependent effects in the ischemic heart [20,21,22]. Functionally, macrophages are broadly classified into two major subtypes: pro-inflammatory and anti-inflammatory [23]. Specifically, pro-inflammatory macrophages exhibit robust expression of inducible nitric oxide synthase (iNOS) and CD86 while actively secreting pro-inflammatory cytokines including interleukin (IL)-1β, IL-6, and tumor necrosis factor (TNF)-α. In contrast, anti-inflammatory macrophages are characterized by high levels of CD206 and are specialized in the production of anti-inflammatory cytokines [24]. Notably, KynA exerts profound modulatory effects on macrophage function, particularly their inflammatory responses, via GPR35 or other receptors [11,13,25].

Based on these observations, we hypothesized that the KynA/GPR35 axis may influence MI by modulating macrophage function. In this study, we demonstrated that KynA promotes cardiac repair by attenuating pro-inflammatory macrophage infiltration and suppressing mitochondrial dysfunction. Mechanistically, activation of GPR35 prevents voltage-dependent anion channel 1 (VDAC1) oligomerization and mitochondrial DNA (mtDNA) leakage, thereby inhibiting the canonical cyclic GMP–AMP synthase (cGAS)/stimulator of interferon genes (STING)/TANK-binding kinase 1 (TBK1)/NF-κB inhibitor α (IκBα) signaling pathway, ultimately resulting in decreased activation of the nuclear factor-κB (NF-κB) P65 subunit (P65) and reduced expression of pro-inflammatory cytokines. Our findings reveal a novel regulatory mechanism that positions KynA/GPR35 signaling as a promising therapeutic target for cardiac repair after MI.

## 2. Materials and Methods

### 2.1. Animals

All animal experiments were performed in compliance with the guidelines of the National Institutes of Health and were approved by the Animal Conservation and Utilization Committee of Huazhong University of Science and Technology. Male C57BL/6J mice (8–12 weeks old) were procured from Vital River Laboratory Animal Technology (Beijing, China).

### 2.2. Animal Models and Treatments

Permanent ligation of the left anterior descending (LAD) coronary artery with 6-0 silk sutures was performed to induce an MI model in mice. Mice in the sham group underwent an identical surgical procedure without ligation of the LAD [26]. KynA (MedChemExpress, Monmouth Junction, NJ, USA) was administered intraperitoneally at 5 mg/kg daily for 5 or 7 consecutive days post-MI [25]. For macrophage depletion, 200 μL of clodronate liposomes (Yeasen, Shanghai, China) were administered intraperitoneally 1 day before MI and again on days 2 and 5 post-MI. Control animals received equivalent volumes of control liposomes [27].

### 2.3. Cell Culture, Transfection and Treatment

Bone marrow-derived macrophages (BMDMs) were generated following a previously described protocol [28]. Briefly, bone marrow cells were flushed from mouse tibiae and femurs, followed by 7 days of culture in DMEM (GIBCO, New York, NY, USA) supplemented with 10% fetal bovine serum (FBS; GIBCO) and 20 ng/mL murine recombinant macrophage colony-stimulating factor (PeproTech, Cranbury, NJ, USA). For pro-inflammatory phenotype polarization, BMDMs were stimulated with 5 ng/mL lipopolysaccharide (LPS; Thermo Fisher Scientific, Waltham, MA, USA) and 12 ng/mL interferon-γ (IFN-γ; R&D Systems, Minneapolis, MN, USA) for 12 h [28]. For functional assays, the cells were treated with 10 mM KynA [19] (MedChemExpress).

GPR35 knockdown was achieved by transfecting cells with small interfering RNA (siRNA; MedChemExpress) using the CALNP RNAi Kit (D-Nano Therapeutics, Beijing, China). The aryl hydrocarbon receptor (AhR) inhibitor CH223191 was applied at a concentration of 10 μM [29].

### 2.4. PKH26 Labeling and Adoptive Transfer of BMDMs

PKH26 (MedChemExpress) was used to label BMDMs according to the manufacturer’s instructions [30]. A total of 2 × 10^6^ PKH26-labeled BMDMs were injected into the tail vein immediately after MI. The mice were then randomized to receive vehicle or KynA treatment for five consecutive days.

### 2.5. RNA Extraction and Real-Time Quantitative Polymerase Chain Reaction (RT-qPCR)

Total RNA was isolated from BMDMs or tissues via TRIzol reagent (Vazyme, Nanjing, China). A NanoDrop 2000 spectrophotometer (Thermo Fisher Scientific) was used to assess RNA concentration and purity. Reverse transcription was performed using PrimeScript RT SuperMix (Vazyme), and qPCR was performed using a SYBR Green Kit (Vazyme) with a Cycler Sequence Detection System (Bio-Rad, Hercules, CA, USA). All primer sequences in our experiments are provided in Supplemental Table S1, and mRNA expression was normalized to *GAPDH* via the 2^−∆∆CT^ method.

### 2.6. Cytosolic mtDNA Quantification

Cytosolic mtDNA was evaluated using a previously described qPCR-based method as described by Aminu et al. [31]. Briefly, total and cytosolic DNA were extracted from BMDMs, and mtDNA abundance was quantified by qPCR using specific primers (Supplemental Table S1).

### 2.7. mtDNA Extraction and Transfection

Mitochondrial DNA was extracted from BMDMs using an mtDNA Extractor Kit (Ab65321, Abcam, Cambridge, MA, USA) following the manufacturer’s instructions. BMDMs were transfected with 5 μg/mL mtDNA for 24 h using Lipofectamine 3000 (Thermo Fisher Scientific).

### 2.8. Flow Cytometry

Infiltrating cardiac immune cells were collected as previously described [26]. Briefly, heart tissues were harvested, minced, and digested with 1 mg/mL collagenase B (Roche, Basel, Switzerland) at 37 °C. Cell suspensions were filtered through 40 μm strainers (BD Biosciences, San Jose, CA, USA) and subjected to flow cytometry analysis. Peripheral blood mononuclear cells (PBMCs) were isolated via Ficoll (Sigma-Aldrich, St. Louis, MO, USA) density gradient centrifugation. Cells were stained with the following antibodies: anti-CD45 FITC (157213; BioLegend, San Diego, CA, USA), anti-CD11b PE-Cy7 (101215; BioLegend), anti-Ly6G PE (127607; BioLegend), anti-CD86 APC-Cy7 (105029; BioLegend), and anti-Ly6C BV711 (755195; BD Biosciences). For intracellular staining, cells were first fixed and permeabilized using corresponding reagents (eBioscience, San Diego, CA, USA), and stained with an anti-F4/80 APC antibody (123116; BioLegend). Cell viability was evaluated via the Zombie Aqua Fixable Viability Kit (423101; BioLegend). Flow cytometry was conducted using a FACSymphony flow cytometer (BD Biosciences).

### 2.9. Echocardiography

Two-dimensional and M-mode echocardiography were performed using a Vevo 2100 imaging system (VisualSonics, Toronto, ON, Canada) 28 days post-MI. Left ventricular ejection fraction (LVEF), left ventricular fractional shortening (LVFS), left ventricular end-diastolic diameter (LVEDD), and left ventricular end-systolic diameter (LVESD) were measured.

### 2.10. 2,3,5-Triphenyltetrazolium Chloride (TTC) Staining

The hearts from MI mice were frozen at −20 °C for 15 min and then cut into 1-mm-thick sections, which were subsequently incubated in 2% 2,3,5-triphenyltetrazolium chloride (TTC) solution (Solarbio, Beijing, China) at 37 °C for 30 min in the dark. After staining, sections were fixed in 4% paraformaldehyde for 40 min. Images were captured using a digital camera, and infarct size was quantified using ImageJ software (version 1.54).

### 2.11. Histology

Hearts were fixed overnight in 4% paraformaldehyde solution, followed by embedding in paraffin and sectioning into serial slices. Scar size and cardiac fibrosis were assessed via Masson’s trichrome staining (Biossci Biotechnology, Beijing, China) and analyzed using Image-Pro Plus software (version 7.0, Media Cybernetics Inc., Bethesda, MD, USA) [26].

### 2.12. Immunofluorescence

Cardiac tissues were fixed in 4% paraformaldehyde for 24 h and embedded in paraffin. Paraffin blocks were then cut into 5 μm-thick sections, which were incubated with primary antibodies against CD68 (14-0681-82, Thermo Fisher) and iNOS (18985-1-AP, Proteintech, Wuhan, China) at 4 °C overnight. DAPI was used to stain the cell nuclei. Images were visualized and captured using a fluorescence microscope (Olympus, Tokyo, Japan).

### 2.13. Western Blotting

To isolate proteins from cells or tissues, RIPA lysis buffer (MedChemExpress) containing a cocktail of protease and phosphatase inhibitors (MedChemExpress) was utilized. Following protein concentration determination with a BCA assay kit (Thermo Fisher Scientific), protein aliquots were separated via sodium dodecyl sulfate–polyacrylamide gel electrophoresis and transferred onto PVDF membranes (Millipore, Billerica, MA, USA). After blocking with buffer (Epizyme Biotech, Shanghai, China), membranes were incubated overnight at 4 °C with the indicated primary antibodies: anti-cGAS (#79978T, Cell Signaling Technology, Danvers, MA, USA), anti-STING (19851-1-AP, Proteintech, Wuhan, China), anti-TBK1(#3504T, Cell Signaling Technology), anti-phospho-TBK1 (#5483T, Cell Signaling Technology), anti-IκBα (#9242S, Cell Signaling Technology), anti-phospho-IκBα (#2859T, Cell Signaling Technology), anti-P65 (#8242T, Cell Signaling Technology), anti-phospho-P65 (#3033T, Cell Signaling Technology), anti-GPR35 (NBP2-24640, Novus Biologicals, Centennial, CO, USA) and anti-β-actin (66009-1-Ig, Proteintech). HRP-conjugated secondary antibodies (Antgene, Wuhan, China) were used for detection, and the blots were visualized using chemiluminescence (Epizyme Biotech) on a ChemiDoc Imaging System (Bio-Rad, Hercules, CA, USA).

### 2.14. VDAC1 Cross-Linking Assay

After PBS (pH 8.0) washing, the BMDMs were incubated with 1 mM cross-linking reagent EGS (Thermo Fisher Scientific) for 30 min. Excess reagent was quenched with Tris buffer (pH 7.5) for 20 min. Cells were lysed, and lysates were analyzed by immunoblotting for VDAC1 (ab154856, Abcam).

### 2.15. Enzyme-Linked Immunosorbent Assay (ELISA)

Following collection of supernatants from BMDM cultures, the concentrations of interleukin-1β (IL-1β), interleukin-6 (IL-6), and tumor necrosis factor-α (TNF-α) were quantified using ELISA kits (Neobioscience, Shenzhen, China) following the manufacturer’s recommended procedures.

### 2.16. Reactive Oxygen Species (ROS), Mitochondrial ROS (mtROS) and Mitochondrial Membrane Potential (MMP) Measurement

ROS levels were evaluated by incubating cultured cells with 2′,7′-dichlorofluorescein diacetate (H_2_DCFDA, Invitrogen, Waltham, MA, USA). Mitochondrial ROS levels were probed using MitoSOX (Invitrogen, USA), and mitochondrial membrane potential (MMP) was assessed using MitoTracker Deep Red (Invitrogen). Fluorescence was analyzed by flow cytometry. For fluorescence assays, cells were incubated with dihydroethidium (DHE, Invitrogen, USA) to evaluate total ROS, MitoSOX (Invitrogen) to detect mtROS, and rhodamine 123 (Rho-123, Invitrogen, USA) to assess MMP. Hoechst 33342 (Beyotime, Shanghai, China) was used for nuclear counterstaining prior to imaging.

### 2.17. RNA Sequencing

BMDMs were subjected to three treatment conditions: LPS/IFN-γ stimulation, LPS/IFN-γ co-stimulation with KynA, and untreated controls (6-h incubation for all groups). Following treatment, cells were harvested, and total RNA was extracted using TRIzol reagent (Invitrogen, CA, USA).

Using the NEBNext UltraTM RNA Library Prep Kit (NEB, Orlando, FL, USA) according to the manufacturer’s instructions, sequencing libraries were prepared as follows: mRNA was isolated from total RNA, fragmented, and converted to cDNA. Post-purification, cDNA was repaired with adapters, PCR-amplified, and re-purified to yield the final library.

After library quality control, raw sequencing data were filtered using SOAPnuke [32] (v2.1.0) to remove: (i) reads containing adapter sequences; (ii) reads with >0.5% ambiguous bases (N); and (iii) low-quality reads (≥50% of bases with a Qphred quality score ≤ 20). Clean reads were aligned to the reference genome (mm10 for mice) using HISAT2 [33] (v2.1.0) and to reference transcripts using Bowtie2 [34] (v2.3.5).

Gene expression levels were quantified using RSEM [35] (v1.3.1) based on Bowtie2 alignments, yielding read counts per transcript, which were normalized to FPKM [36] (Fragments Per Kilobase per Million bases). Using DESeq235 [37] (v1.22.2), we performed differential expression analysis; significant DEGs were identified based on FDR < 0.05 and |log_2_fold change| > 1. Biological functions of these DEGs were annotated via Gene Ontology (GO) and Kyoto Encyclopedia of Genes and Genomes (KEGG) pathway enrichment analyses.

### 2.18. Statistical Analysis

The Shapiro–Wilk test was used to assess normality, while the F-test (for 2 groups) or Bartlett’s test (for multiple groups) was employed to verify variance homogeneity. For normally distributed data with homogeneous variance, group differences were analyzed using the unpaired Student’s *t*-test (2 groups) or one- or two-way ANOVA with Tukey’s post hoc test (≥3 groups). Non-parametric comparisons employed the Mann–Whitney U test (2 groups) or Kruskal–Wallis test with Dunn’s post hoc test (≥3 groups). All analyses were run in GraphPad Prism 10.1.1, with the significance threshold set at *p* < 0.05.

## 3. Results

### 3.1. KynA Enhances Cardiac Repair After MI

**Figure 1:**
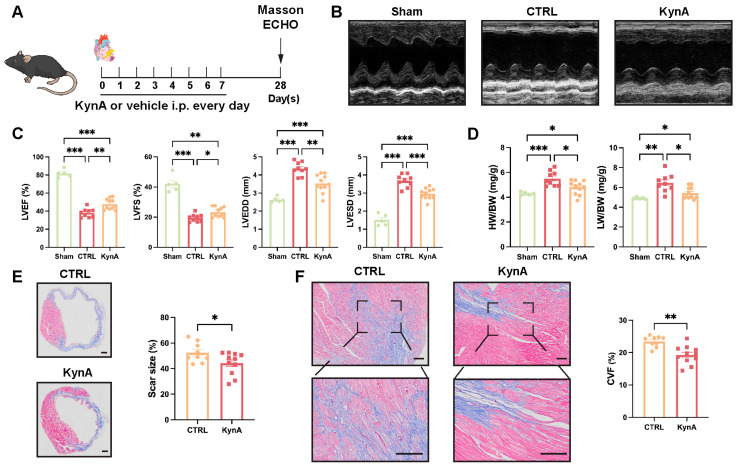
KynA attenuates cardiac dysfunction after MI. (**A**) Experimental design. Sham-operated mice received vehicle alone for 7 days, while MI mice were administered intraperitoneal KynA or vehicle for 7 days post-MI. Echocardiography and Masson’s trichrome staining were performed at day 28. (**B**) Representative M-mode echocardiogram images acquired at 28 days post-MI. (**C**) Echocardiographic analysis of LVEF, LVFS, LVEDD, and LVESD was performed 28 days post-MI. *n* = 5–11 per group. (**D**) HW/BW and LW/HW ratios were determined 28 days post-MI. *n* = 5–11 per group. (**E**) Representative images (**Left**) and quantitative analysis (**Right**) of infarct scar size using Masson’s trichrome staining. Scale bar: 500 μM. *n* = 9–11 per group. (**F**) Representative images (**Left**) and quantitative analysis (**Right**) of cardiac fibrosis, as assessed by Masson’s trichrome staining, 28 days post-MI. Scale bar: 100 μm. *n* = 9–11 per group. Statistical comparisons: one-way ANOVA was performed in (**C**,**D**); 2-tailed unpaired *t*-test was performed in (**E**,**F**). * *p* < 0.05, ** *p* < 0.01, *** *p* < 0.001. KynA, Kynurenic Acid; LVEF, left ventricular ejection fraction; LVFS, left ventricular fractional shortening; LVEDD, left ventricular end-diastolic dimension; LVESD, left ventricular end-systolic dimension; HW, heart weight; BW, body weight; LW, lung weight; CVF, collagen volume fraction.

To confirm the consistency of the surgical procedure, TTC staining was performed to assess myocardial infarct size at 24 h after LAD ligation. The comparable infarct sizes observed verified the uniformity of the surgical operation (Figure S1A,B). To assess the effect of KynA on cardiac function post-MI, mice received KynA intraperitoneally for 7 days following LAD ligation, and left ventricular function was examined 4 weeks later (Figure 1A). KynA treatment markedly improved LVEF and LVFS while attenuating LVEDD and LVESD (Figure 1B,C). Furthermore, KynA treatment failed to fully restore cardiac function, as reflected by the persistent difference between the KynA-treated and sham-operated groups (Figure 1B,C). Consistently, both the heart weight-to-body weight ratio (HW/BW) and lung weight-to-body weight ratio (LW/BW) were decreased in KynA-treated mice (Figure 1D). Masson’s trichrome staining revealed significant reductions in scar size and interstitial fibrosis (Figure 1E,F). Collectively, these findings demonstrate that KynA promotes post-infarction cardiac repair, attenuates adverse remodeling, and preserves cardiac function.

### 3.2. KynA Suppresses Recruitment of Proinflammatory Macrophages After MI

**Figure 2:**
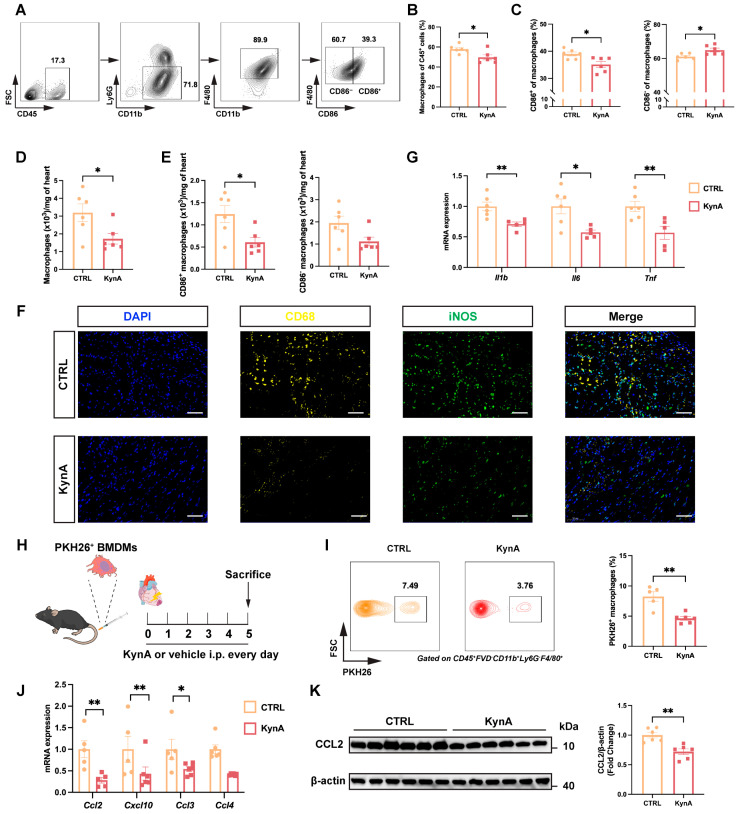
KynA attenuates proinflammatory macrophage population recruitment in the heart following MI. (**A**–**E**) Mice received daily vehicle or KynA treatment after MI. The animals were sacrificed on day 5 after MI and heart tissues were harvested for flow cytometric analysis. *n* = 6 per group. (**A**) The gating strategy of macrophages in the hearts. (**B**) Proportions of macrophages in the hearts of MI mice treated with vehicle or KynA. (**C**) Proportions of CD86^+^ (**Left**) and CD86^−^ (**Right**) macrophages in the hearts of MI mice treated with vehicle or KynA. (**D**) Quantification of cardiac infiltrating macrophages post-MI. (**E**) Quantification of cardiac infiltrating subsets of CD86^+^ (**Left**) and CD86^−^ (**Right**) macrophages post-MI. (**F**) Immunofluorescence co-staining for CD68 (yellow) and iNOS (green), scale bar = 50 μm, *n* = 5 per group. (**G**) *Il1b*, *Il6* and *Tnf* mRNA expression levels in the hearts of mice treated with vehicle or KynA. *n* = 5–6 per group. (**H**) Experimental design. After MI, mice received PKH26-labelled BMDMs. The mice were then randomised to receive vehicle or KynA treatment for 5 days. (**I**) Representative flow cytometry plots (**Left**) and proportions (**Right**) of PHK26^+^ macrophages in the heart. *n* = 5–6 per group. (**J**) The mRNA expression levels of *Ccl2*, *Cxcl10*, *Ccl3*, and *Ccl4* in the hearts of mice treated with vehicle or KynA. *n* = 5–6 per group. (**K**) Representative Western blotting images (**Left**) and quantification (**Right**) of Ccl2 protein expression levels in the hearts of mice treated with vehicle or KynA. *n* = 6 per group. Statistical comparisons: 2-tailed unpaired *t*-test was performed in (**B**–**E**,**G**,**I**–**K**). * *p* < 0.05, ** *p* < 0.01. *Il1b*, Interleukin 1β; *Il6*, Interleukin 6; *Tnf*, Tumor necrosis factor; BMDMs, Bone marrow-derived macrophages; *Ccl2*, C-C motif chemokine ligand 2; *Cxcl10*, C-X-C motif chemokine 10; *Ccl3*, C-C motif chemokine ligand 3; *Ccl4*, C-C motif chemokine ligand 3.

KynA has been implicated as an immunomodulator with prominent effects on macrophages [11,12,38]. Therefore, we first investigated whether the exogenous administration of KynA had an effect on the number and proportion of macrophages and their subtypes in the heart after MI. Flow cytometry analysis revealed a significantly reduced proportion of total macrophages in KynA-treated hearts (Figure 2A,B). Subset analysis demonstrated a marked decline in pro-inflammatory macrophages and a relative increase in anti-inflammatory macrophages (Figure 2C). Absolute quantification revealed a statistically significant reduction in total macrophages (Figure 2D), driven exclusively by a decrease in the pro-inflammatory subset; anti-inflammatory macrophages did not differ significantly from controls (Figure 2E). Immunofluorescent staining validated the observed reduction in macrophages, particularly pro-inflammatory macrophages (Figure 2F). Consistent with these findings, KynA treatment led to a significant decrease in the expression of IL-1β, IL-6, and TNF-α in the heart (Figure 2G).

Subsequently, to investigate whether KynA influences monocyte infiltration into the myocardium, we analyzed circulating monocyte populations post-MI. Our findings revealed no statistically significant differences in the frequency or absolute counts of total monocytes or in the Ly6C^hi^ and Ly6C^lo^ subsets (Figure S2A–D). We then performed adoptive transfer experiments to directly assess monocyte recruitment. BMDMs were labeled with PKH26, a fluorescent cell membrane linker dye widely used for live cell tracking [30]. PKH26-labeled BMDMs were intravenously administered to mice after MI (Figure 2H). Mice in the KynA group exhibited significantly reduced accumulation of PKH26-positive macrophages in the infarcted heart (Figure 2I). Macrophage-associated chemokines, including C-X-C motif chemokine 10 (Cxcl10), C-C motif chemokine ligand 2 (*Ccl2*), C-C motif chemokine ligand 3 (*Ccl3*), and C-C motif chemokine ligand 4 (*Ccl4*), were also assessed. KynA downregulated the expression levels of *Cxcl10*, *Ccl2*, and *Ccl3* mRNA in infarcted hearts (Figure 2J). We also detected CCL2 protein, a key chemokine in macrophages, and found that KynA administration decreased CCL2 protein abundance (Figure 2K). Together, these data indicate that KynA selectively suppresses chemokine-driven recruitment of pro-inflammatory macrophages into the injured myocardium.

### 3.3. Depletion of Macrophages Abrogates the Protective Effects of KynA

**Figure 3:**
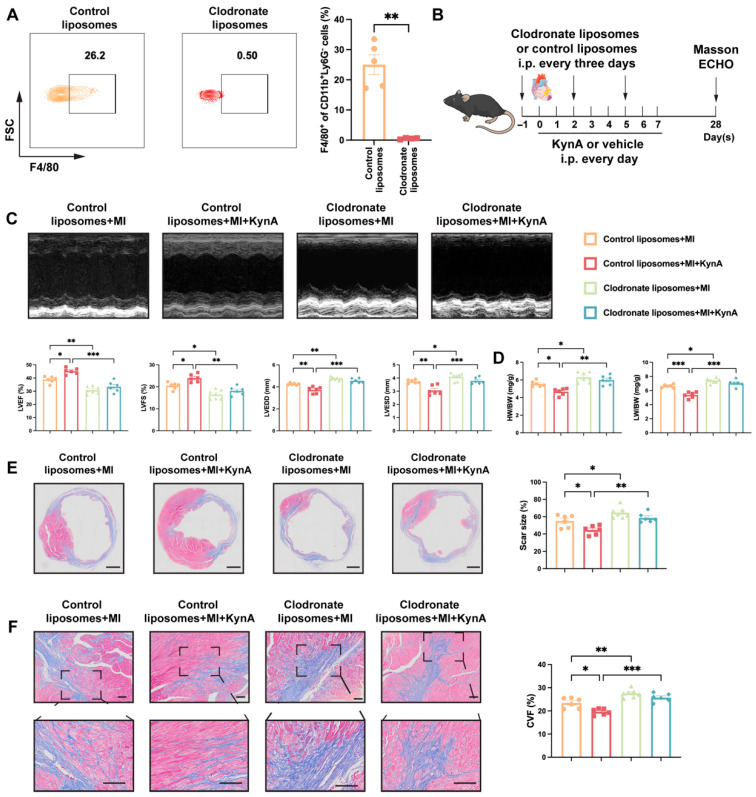
The depletion of endogenous macrophages diminishes the protective effects of KynA. (**A**) Mice received intraperitoneal administration of clodronate liposomes or control liposomes. After 3 days, flow cytometry was applied to confirm systemic macrophage depletion. Representative flow cytometry plots (**Left**) and percentages (**Right**) of F4/80^+^ macrophages in the spleens of mice. *n* = 4–5 per group. (**B**) Experimental design. Mice received intraperitoneal administration of clodronate liposomes on day -1 before MI and days 2 and 5 after MI to deplete macrophages; control liposomes were used as the control. Mice received intraperitoneal injections of KynA or vehicle for 8 consecutive days after MI. Echocardiography and Masson’s trichrome staining were performed at day 28. (**C**) Representative echocardiogram images in M-mode (**Top**) and echocardiographic assessment of LVEF, LVFS LVEDD, and LVESD (**Bottom**) at 28 days after MI. *n* = 6–7 per group. (**D**) HW/BW and LW/HW ratios determined at 28 days after MI. *n* = 6–7 per group. (**E**) Representative images (**Left**) and quantitative analysis (**Right**) of infarct scar size using Masson’s trichrome staining. Scale bar: 1 mm. *n* = 6–7 per group. (**F**) Representative images (**Left**) and quantitative analysis (**Right**) of cardiac fibrosis using Masson’s trichrome staining at 28 days after MI. Scale bar: 100 μm. *n* = 6–7 per group. Statistical comparisons: 2-tailed unpaired *t*-test was performed in (**A**); two-way ANOVA was performed in (**C**–**F**). * *p* < 0.05, ** *p* < 0.01, *** *p* < 0.001.

To further investigate whether KynA exerts a protective effect against MI through macrophages, clodronate liposomes were intraperitoneally injected to deplete macrophages, and successful systemic depletion was confirmed 3 days after administration (Figure 3A). The mice received clodronate liposomes 1 d before MI and on post-MI days 2 and 5 [27]. KynA was administered immediately after MI induction and once daily thereafter (Figure 3B). Consistent with previous findings, KynA improved cardiac function and reduced cardiac dimensions in post-MI mice. However, the cardioprotective effects of KynA were abolished upon macrophage depletion (Figure 3C). Similarly, the HW/BW and LW/BW ratios, scar size, and fibrosis did not show any difference between the KynA and control groups under macrophage-depleted conditions (Figure 3D–F). Consistent with previous studies [39,40], clodronate liposome treatment impaired wound healing. Collectively, these results demonstrate that macrophages are indispensable effectors through which KynA exerts its cardioprotective effects.

### 3.4. KynA/GPR35 Signaling Inhibits Pro-Inflammatory Macrophage Polarization by Improving Mitochondrial Function

**Figure 4:**
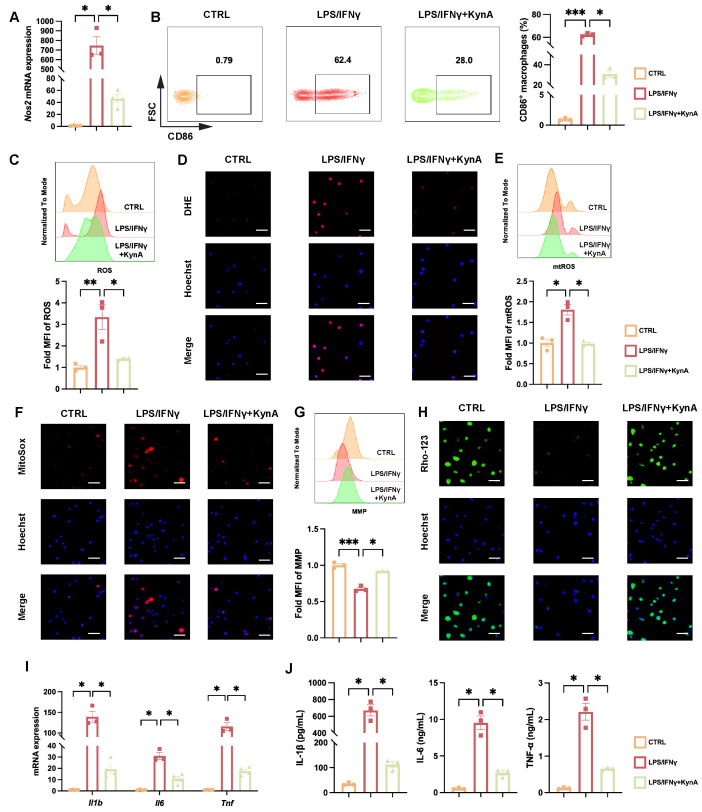
KynA attenuates the proinflammatory macrophage phenotype. BMDMs were collected after being treated with vehicle, LPS/IFNγ, or 10 mM KynA in combination with LPS/IFNγ for 12 h. (**A**) The mRNA expression of *Nos2* across the experimental groups. *n* = 3–4 per group. (**B**) Representative flow cytometry plots (**Left**) and percentages (**Right**) of CD86^+^ macrophages among the indicated groups. *n* = 3 per group. (**C**) Representative flow cytometry histograms (**Top**) and MFI (**Bottom**) of ROS levels across the experimental groups. *n* = 3 per group. (**D**) Representative immunofluorescence images of DHE staining. Scale bar: 50 μm. *n* = 3 per group. (**E**) Representative flow cytometry histograms (**Top**) and MFI (**Bottom**) of mtROS levels across the experimental groups. *n* = 3 per group. (**F**) Representative immunofluorescence images of MitoSox staining. Scale bar: 50 μm. *n* = 3 per group. (**G**) Representative flow cytometry histograms (**Top**) and (**Bottom**) of MMP levels across the experimental groups. n = 3 per group. (**H**) Representative immunofluorescence images of Rho-123 staining. Scale bar: 50 μm. *n* = 3 per group. (**I**) *Il1b*, *Il6* and *Tnf* mRNA expression levels among the indicated groups. *n* = 3–4 per group. (**J**) ELISA quantification of IL-1β, IL-6, and TNF-α secretion in cell culture supernatants. *n* = 3 per group. Statistical comparisons: one-way ANOVA was performed in (**A**–**C**,**E**,**G**,**I**,**J**). * *p* < 0.05, ** *p* < 0.01, *** *p* < 0.001. LPS/IFNγ, Lipopolysaccharide/Interferon γ; iNOS, Inducible nitric oxide synthase; ROS, Reactive oxygen species; MFI, mean fluorescence intensity; DHE, Dihydroethidium; mtROS, Mitochondrial ROS; MMP, Mitochondrial membrane potential; Rho-123, Rhodamine 123; TNF-α, Tumor necrosis factor α.

Next, we investigated whether KynA exerts a direct regulatory effect on macrophage polarization and favors the anti-inflammatory over the pro-inflammatory phenotype. We employed LPS and IFN-γ to induce polarization of BMDMs into pro-inflammatory macrophages while adding KynA or vehicle [28]. Our results revealed that KynA treatment significantly downregulated Nos2 expression (Figure 4A), and decreased the proportion of CD86^+^ pro-inflammatory macrophages (Figure 4B). These results suggest that KynA inhibits macrophage differentiation into pro-inflammatory subtypes. Recently, several studies show KynA prevents ATP hydrolysis and decreases mitochondrial damage, affecting mitochondrial function [7,19], which, along with ROS generation, is pivotal for pro-inflammatory macrophage function including signal transduction, redox reactions and autophagy [41,42]. Therefore, we assessed cellular ROS levels and mitochondrial homeostasis. KynA treatment reduced both total and mitochondrial ROS while enhancing MMP, as shown by flow cytometry and confirmed by immunofluorescence imaging (Figure 4C–H). Consistently, the transcription and secretion of IL-1β, IL-6, and TNF-α were significantly reduced (Figure 4I,J). Thus, KynA suppresses the pro-inflammatory phenotype of macrophages and preserves mitochondrial function.

**Figure 5:**
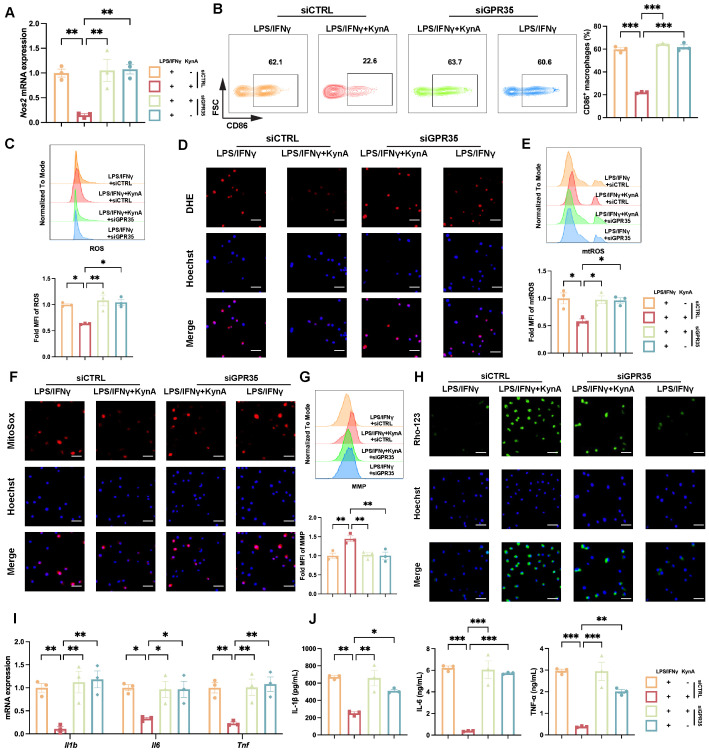
KynA inhibits proinflammatory macrophages via GPR35. BMDMs were transfected with either control siRNA or GPR35 siRNA. Subsequently, the cells were treated with either LPS/IFNγ alone or 10 mM KynA administered in combination with LPS/IFNγ for a duration of 12 h. (**A**) The mRNA expression of *Nos*2 across the experimental groups. *n* = 3 per group. (**B**) Representative flow cytometry plots (**Left**) and percentages (**Right**) of CD86^+^ macrophages among the indicated groups. *n* = 3 per group. (**C**) Representative flow cytometry histograms (**Top**) and MFI (**Bottom**) of ROS levels across the experimental groups. *n* = 3 per group. (**D**) Representative immunofluorescence images of DHE staining. Scale bar: 50 μm. *n* = 3 per group. (**E**) Representative flow cytometry histograms (**Top**) and MFI (**Bottom**) of mtROS levels across the experimental groups. *n* = 3 per group. (**F**) Representative immunofluorescence images of MitoSox staining. Scale bar: 50 μm. *n* = 3 per group. (**G**) Representative flow cytometry histograms (**Top**) and MFI (**Bottom**) of MMP levels across the experimental groups. *n* = 3 per group. (**H**) Representative immunofluorescence images of Rho-123 staining. Scale bar: 50 μm. *n* = 3 per group. (**I**) *Il1b*, *Il6* and *Tnf* mRNA expression levels among the indicated groups. *n* = 3 per group. (**J**) ELISA quantification of IL-1β, IL-6, and TNF-α secretion in cell culture supernatants. *n* = 3 per group. Statistical comparisons: two-way ANOVA was performed in (**A**–**C**,**E**,**G**,**I**,**J**). * *p* < 0.05, ** *p* < 0.01, *** *p* < 0.001. GPR35, G protein-coupled receptor 35.

As KynA can signal via multiple receptors, including GPR35, ionotropic glutamate receptors, and the aryl hydrocarbon receptor (AhR) [19,25,43,44], we aimed to determine which receptor mediates the anti-inflammatory effects of KynA in BMDMs. Given that ionotropic glutamate receptors are predominantly involved in neurotransmission [19], we hypothesized that KynA exerts its effects via AhR or GPR35. First, we used CH223191 [29], an AhR inhibitor (AhRi), to confirm if AhR mediates KynA’s anti-inflammatory actions. However, our results showed that blocking AhR did not alter the effects of KynA in BMDMs (Figure S3A). Subsequently, small interfering RNA (siRNA) was transfected to knock down GPR35 expression of BMDMs, which was confirmed by a significant reduction in protein levels (Figure S3B). GPR35 knockdown reversed KynA-mediated inhibition of pro-inflammatory macrophage polarization by restoring *Nos2* expression and the proportion of CD86^+^ cells (Figure 5A,B), increasing ROS and mtROS levels with concomitant MMP reduction (Figure 5C–H), and elevating pro-inflammatory cytokine levels (Figure 5I–J). These findings demonstrate that the anti-inflammatory effect of KynA is predominantly mediated by GPR35.

### 3.5. KynA/GPR35 Signaling Prevents mtDNA Leakage and Suppresses cGAS/STING Activation

**Figure 6:**
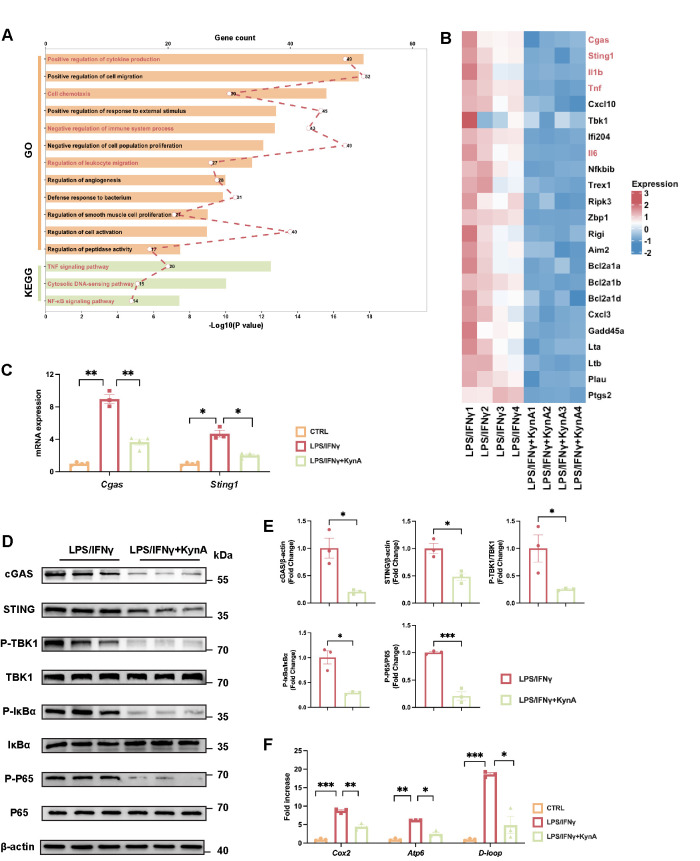
KynA-mediated inhibition of mtDNA leakage results in downregulation of the cGAS/STING pathway. (**A**,**B**) BMDMs were treated with either LPS/IFNγ alone or with 10 mM KynA in combination with LPS/IFNγ for 12 h. The BMDMs were harvested for RNA sequencing (RNA-seq) analysis after treatment. *n* = 4 per group. (**A**) Enrichment analysis of downregulated genes in the LPS/IFNγ + KynA treated group versus the LPS/IFNγ group. (**B**) Heatmap of selected enriched pathways based on downregulated genes in the LPS/IFNγ + KynA-treated group compared to the LPS/IFNγ group. (**C**) The mRNA expression of *Cgas* and *Sting*1 among the indicated groups. *n* = 3–4 per group. (**D**) Representative Western blotting images of the indicated protein expression levels across experimental groups. *n* = 3 per group. (**E**) Quantification of the indicated protein expression levels across experimental groups. *n* = 3 per group. (**F**) Quantitative analysis of mtDNA content, including *Cox2*, *Atp6*, and *D-loop* regions, across the experimental groups. *n* = 3 per group. Statistical comparisons: 2-tailed unpaired *t*-test was performed in (**E**); one-way ANOVA was performed in (**C**,**F**). * *p* < 0.05, ** *p* < 0.01, *** *p* < 0.001. GO, Gene Ontology; KEGG, Kyoto Encyclopedia of Genes and Genomes; cGAS, Canonical cyclic GMP-AMP synthase; STING, Stimulator of interferon genes; TBK1, TANK-binding kinase 1; IκBα, NF-κB inhibitor α; P65, Nuclear factor-κB P65 subunit; mtDNA, Mitochondrial DNA; Cox2, Cytochrome c oxidase II; Atp6, ATP synthase membrane subunit 6; D-loop, D-loop region.

To delineate the molecular mechanisms downstream of KynA/GPR35, RNA sequencing (RNA-seq) was performed on LPS/IFN-γ-stimulated BMDMs treated with or without KynA. Principal component analysis (PCA) uncovered clear differences in transcriptional profiles between the two groups. (Figure S4A). Compared with the LPS/IFN-γ-treated group, KynA markedly altered the gene expression profile, as shown in the heatmap (Figure S4B). Specifically, 357 genes were upregulated and 679 genes were downregulated following KynA treatment (Figure S4C). Enrichment analysis of downregulated genes indicated significant suppression of pathways related to cytokine production and leukocyte migration and, notably, the cytosolic DNA-sensing and NF-κB pathways (Figure 6A). Specifically, KynA treatment reduced the expression of *Cgas*, *Sting1*, and multiple inflammatory mediators (*Il1b*, *Tnf*, and *Il6*6) (Figure 6B). Previous work has elucidated that the cGAS/STING signaling pathway governs proinflammatory cytokine production by activating downstream effectors, such as TBK1 and NF-κB, through phosphorylation events [45,46,47]. Thus, we confirmed decreased *Cgas* and *Sting1* expression after KynA treatment (Figure 6C). Immunoblotting corroborated these findings, showing reduced protein abundance of cGAS and STING and decreased phosphorylation of TBK1, IκBα, and P65 following KynA treatment (Figure 6D,E).

**Figure 7:**
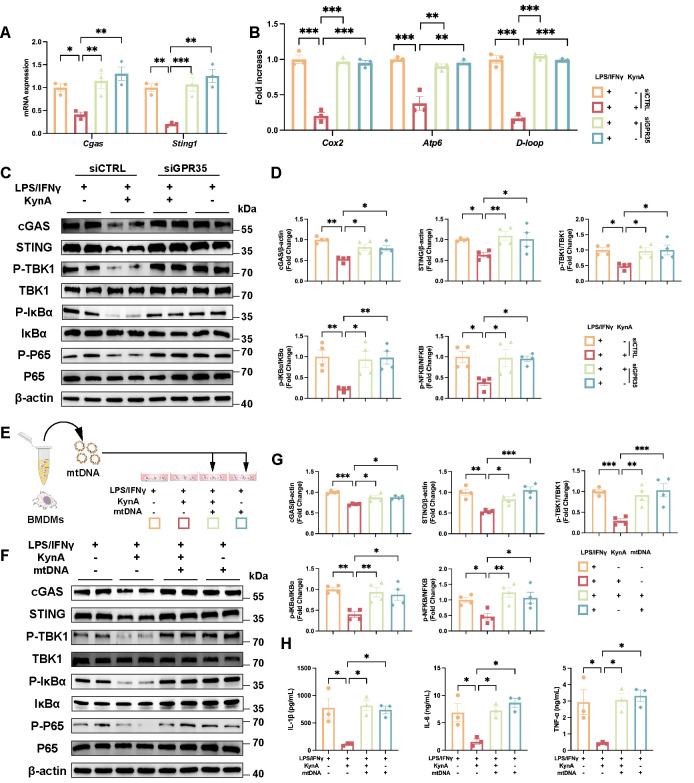
KynA suppresses the cGAS/STING pathway through GPR35-mediated inhibition of mtDNA leakage in macrophages. (**A**) The mRNA expression of *Cgas* and *Sting1* was analyzed across the experimental groups. *n* = 3 per group. (**B**) Quantitative assessment of mtDNA content, including *Cox2*, *Atp6*, and *D-loop* regions, among the experimental groups. (**C**) Representative immunoblotting images of the indicated protein across the experimental groups. *n* = 4 per group. (**D**) Quantification analysis of the indicated protein across the experimental groups. *n* = 4 per group. (**E**) Schematic illustration of the experimental design for mtDNA stimulation in BMDMs. The mtDNA was extracted and transfected into BMDMs with 5 μg/mL after treated with either LPS/IFNγ alone or with KynA in combination with LPS/IFNγ. (**F**) Representative Western blotting images of the indicated protein across the experimental groups. *n* = 4 per group. (**G**) Quantification analysis of the indicated protein across the experimental groups. *n* = 4 per group. (**H**) ELISA quantification of IL-1β, IL-6, and TNF-α secretion in cell culture supernatants among indicated groups. *n* = 3 per group. Statistical comparisons: two-way ANOVA was performed in (**A**,**B**,**D**,**G**,**H**). * *p* < 0.05, ** *p* < 0.01, *** *p* < 0.001.

Given that mitochondrial damage is a well-documented trigger of cytosolic mtDNA release and subsequent cGAS activation [48,49,50], we evaluated mtDNA leakage. KynA significantly reduced cytosolic mtDNA abundance, as demonstrated by lower levels of cytochrome c oxidase II (*Cox2*), ATP synthase membrane subunit 6 (*Atp6*), and mitochondrial D-loop region (*D-loop*) sequences (Figure 6F). In addition, knockdown of GPR35 significantly reversed the inhibitory effect of KynA on mitochondrial dysfunction, as evidenced by upregulation of *Cgas*, *Sting1* expression and increased mtDNA leakage (Figure 7A,B). Immunoblotting further confirmed that GPR35 knockdown reversed the inhibitory effects of KynA on cGAS, STING, TBK1, IκBα, and P65 activation (Figure 7C,D).

To directly determine whether KynA exerts its effects through inhibition of mtDNA release, BMDMs were transfected with exogenous mtDNA, as previously described [49] (Figure 7E). The addition of mtDNA completely counteracted the inhibitory actions of KynA, reactivating the cGAS/STING pathway and restoring pro-inflammatory cytokine production (Figure 7F–H). These data indicate that prevention of mtDNA leakage and downstream cGAS/STING activation are crucial mechanisms underlying the anti-inflammatory action of the KynA/GPR35 axis.

### 3.6. KynA/GPR35 Inhibits mtDNA Leakage by Suppressing VDAC1 Oligomerization

**Figure 8:**
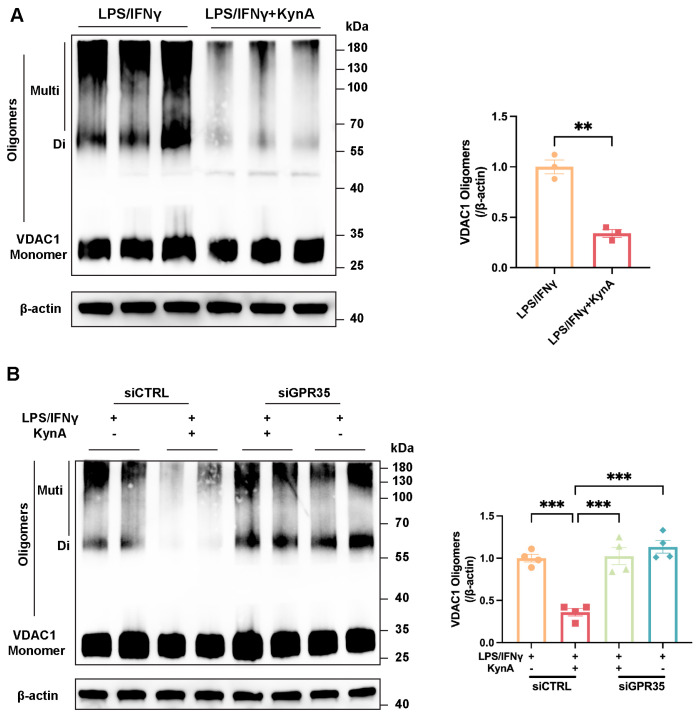
KynA/GPR35-mediated VDAC1 oligomerization controls cytosolic mtDNA release in macrophages. (**A**) Representative immunoblot images (**Left**) and corresponding quantitative analysis (**Right**) of VDAC1 expression across the experimental groups. *n* = 3 per group. (**B**) Representative immunoblot images (**Left**) and corresponding quantitative analysis (**Right**) of VDAC1 expression across the experimental groups. *n* = 4 per group. Statistical comparisons: 2-tailed unpaired *t*-test was performed in (**A**); two-way ANOVA was performed in (**B**). ** *p* < 0.01, *** *p* < 0.001. VDAC1, Voltage-dependent anion channel 1; Di, Dimers; Muti, multimers.

Finally, we investigated the mechanism through which the KynA/GPR35 axis prevents mtDNA leakage. VDAC1, a mitochondrial outer membrane protein, forms oligomeric pores that facilitate mtDNA release during stress and inflammation [51,52]. Immunoblot analysis revealed that KynA treatment markedly reduced VDAC1 oligomerization, which correlated with reduced mtDNA release (Figure 8A). Importantly, this effect was reversed by GPR35 knockdown (Figure 8B). These results suggest that the KynA/GPR35 axis inhibits activation of the cGAS/STING pathway by limiting VDAC1-mediated mtDNA leakage in macrophages.

## 4. Discussion

This study revealed that the KynA/GPR35 signaling axis is an important regulator of post-MI cardiac repair. We showed that activation of this pathway suppressed infiltration and pro-inflammatory polarization of cardiac macrophages, preserved mitochondrial integrity, and attenuated VDAC1-dependent mtDNA leakage. This prevents aberrant activation of the cGAS/STING/TBK1/IκBα/P65 signaling cascade, reduces inflammatory cytokine production, and ultimately improved ventricular remodeling and function. These findings expand the current understanding of KynA/GPR35 axis biology by linking it to macrophage mitochondrial function, thereby identifying a potential therapeutic target for acute myocardial infarction.

Our results demonstrated that KynA plays a protective role in promoting cardiac recovery subsequent to MI. To date, most previous work has centered on the role of KynA on myocardial ischemia/reperfusion injury (MIRI), demonstrating that KynA protects cardiomyocytes against apoptosis and preserves their mitochondrial function [19,53,54,55,56]. Our findings confirmed that KynA improves MI by regulating immune cell function. Macrophage depletion experiments demonstrated that the protective effect of KynA on ventricular remodeling after MI was primarily mediated through macrophages. However, we still cannot exclude the potential impact of KynA on cardiomyocytes in MI, given the documented protection role of KynA on cardiomyocytes in MIRI. The biological effects of KynA may depend on the severity of the infarction, as well as the concentration and administration duration of KynA. Further studies are needed to clarify this issue. Furthermore, we also found that KynA reduced cardiac macrophage infiltration and inhibit the polarization of pro-inflammatory macrophages. Specifically, we demonstrate decreased levels of chemokines related to macrophage migration such as CCL2. However, a recent study reported that KynA recruits GPR35^+^ macrophages to promote experimental encephalitis [11].

In addition, we show that the KynA/GRP35 axis reduces pro-inflammatory cytokine production (IL-1β, IL-6, and TNF-α) and alleviates oxidative stress by lowering both cellular and mitochondrial ROS levels. These effects parallel those of earlier reports in which KynA suppressed TNF-α and IL-1β expression and mitigated inflammation in models of colitis [8,9].

Notably, GPR35 has multiple ligands beyond KynA, including C-X-C motif chemokine ligand 17 (Cxcl17), lysophosphatidic acid (LPA), and 5-hydroxyindoleacetic acid (5-HIAA) [57]. Previous studies showed that activation of GPR35 by these ligands promotes leukocyte recruitment in inflamed tissues [11,15,57]. Interestingly, our study demonstrated the opposite effect in the heart, with KynA/GPR35 signaling reducing macrophage accumulation. These contrasting outcomes emphasize the complexity of GPR35 signaling and the importance of tissue-specific microenvironments in determining functional outputs.

Mechanistically, our findings suggest that KynA/GPR35 may stabilize mitochondrial function by inhibiting the oligomerization of VDAC1, a mitochondrial outer membrane protein. As a previous study demonstrated that GPR35 can traffic to the outer mitochondria membrane and interact with mitochondrial proteins19, we hypothesize that GPR35 may also form an interaction with VDAC1 at the outer mitochondrial membrane. However, further studies are required to validate this potential interaction and clarity its detailed underlying mechanism. Because mtDNA also activates multiple danger-sensing pathways beyond cGAS/STING, including Toll-like receptor 9 (TLR9) and the nucleotide-binding domain leucine-rich repeat protein 3 (NLRP3) inflammasome [51], it is plausible that the KynA/GPR35 axis exerts broader immunomodulatory effects. Future studies should extend this investigation by including additional innate immune checkpoints and cardiac immune cell populations.

In conclusion, our study established that the KynA/GPR35 axis is a pivotal regulator of post-MI inflammation. By limiting VDAC1-mediated mtDNA release and inhibiting downstream inflammatory signaling in macrophages, this axis promotes cardiac repair in injured hearts. These findings not only provide mechanistic insights into the immunoregulatory actions of the KynA/GRP35 axis but also highlight a promising therapeutic avenue for attenuating deleterious inflammation and improving outcomes in ischemic heart disease.

## Data Availability

The raw RNA sequencing (RNA-seq) data reported in this study were deposited in the NCBI Sequence Read Archive (SRA) repository under accession number SRR36968683, SRR36968684, SRR36968685, SRR36968686, SRR36968687, SRR36968688, SRR36968689 and SRR36968690. All other relevant data supporting the findings of this study are included in the article and its Supplementary Materials.

## Supplementary Materials

The following supporting information can be downloaded at: https://www.mdpi.com/article/10.3390/antiox15030300/s1.

## Abbreviations

TTC	2,3,5-triphenyltetrazolium chloride
H_2_DCFDA	2′,7′-dichlorofluorescein diacetate
5-HIAA	5-hydroxyindoleacetic acid
AhRi	AhR inhibitor
AhR	Aryl hydrocarbon receptor
*Atp6*	ATP synthase membrane subunit 6
BMDMs	Bone marrow-derived macrophages
cGAS/*Cgas*	Canonical cyclic GMP-AMP synthase
CCL2/*Ccl2*	C-C motif chemokine ligand 2
*Ccl3*	C-C motif chemokine ligand 3
*Ccl4*	C-C motif chemokine ligand 4
*Cxcl10*	C-X-C motif chemokine 10
*Cxcl17*	C-X-C motif chemokine ligand 17
*Cox2*	Cytochrome c oxidase II
DEGs	Differentially expressed genes
DHE	Dihydroethidium
ELISA	Enzyme-Linked Immunosorbent Assay
GPR35	G protein-coupled receptor 35
HW/BW	Heart weight to body weight ratio
iNOS/*Nos2*	Inducible nitric oxide synthase
IFN-γ	Interferon-γ
IL-1β/*Il1b*	Interleukin-1β
IL-6/*Il6*	Interleukin-6
KynA	Kynurenic acid
LAD	Left anterior descending
LVESD	Left end-systolic diameters
LVEF	Left ventricular ejection fraction
LVEDD	Left ventricular end-diastolic
LVFS	Left ventricular fractional shortening
LPS	Lipopolysaccharide
LW/BW	Lung weight to body weight ratio
LPA	Lysophosphatidic acid
*D-loop*	Mitochondrial D-loop region
mtDNA	Mitochondrial DNA
MMP	Mitochondrial membrane potential
mtROS	Mitochondrial ROS
MI	Myocardial infarction
IκBα	NF-κB inhibitor α
NF-κB	Nuclear factor-κB
P65	Nuclear factor-κB P65 subunit
NLRP3	Nucleotide-binding domain and leucine-rich repeat protein-3
PCA	Principal component analysis
ROS	Reactive oxygen species
RT-qPCR	Real-time quantitative polymerase chain reaction
Rho-123	Rhodamine 123
RNA-seq	RNA sequencing
siRNA	Small interfering RNA
STING/*Sting1*	Stimulator of interferon genes
TBK1	TANK-binding kinase 1
TLR9	Toll-like receptor 9
TNF/*Tnf*	Tumor necrosis factor
VDAC1	Voltage-dependent anion channel 1
