# Supplementary material for: Kynurenic Acid/GPR35 Signaling Protects the Infarcted Heart by Suppressing Macrophage mtDNA-Triggered cGAS-STING Activation
Source: Antioxidants (Basel). 2026 Feb 27;15(3):300. doi: 10.3390/antiox15030300 (PMC13024547; doi:10.3390/antiox15030300)
Supplement: Supplementary file 1 [file antioxidants-15-00300-s001.zip › antioxidants-4125455-supplementary.pdf]

Supplementary Materials

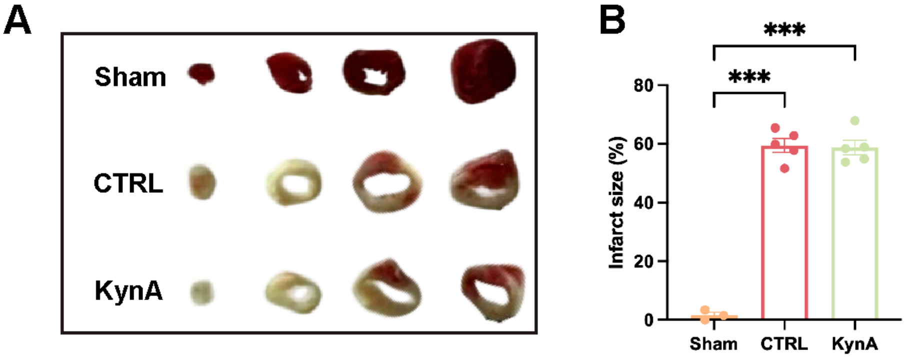

Figure S1. Quantification of myocardial infarct size.

Mice received daily vehicle or KynA treatment following MI. Animals were euthanized at day 5 post-MI, and peripheral blood were harvested for flow cytometric analysis. n = 6 per group.

A. Representative cardiac sections stained with TTC.

B. Quantitative analysis of myocardial infarct size. n = 3-5 per group.

Statistical comparisons: one-way ANOVA was performed in (B). *** $P < 0.001$.

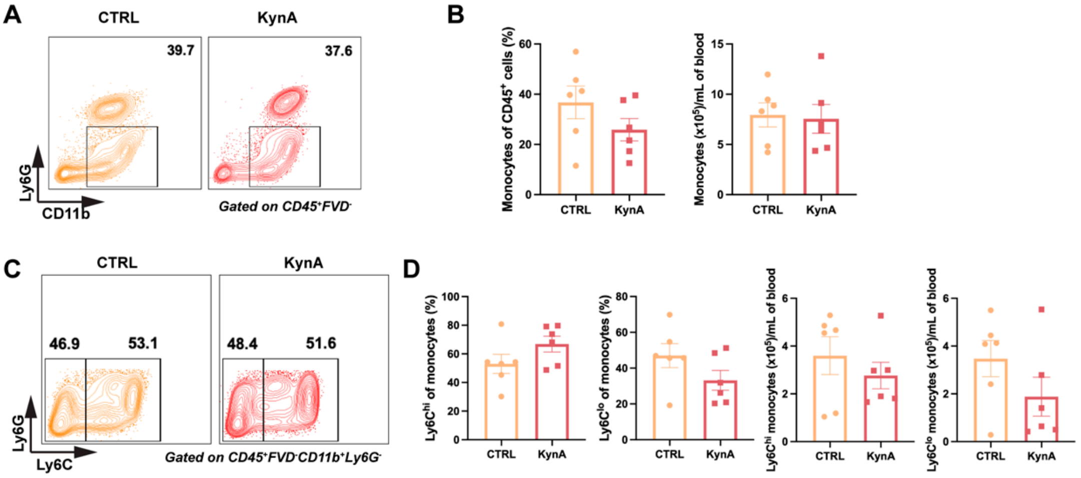

Figure S2. The peripheral blood monocytes show no difference in MI mice treated with or without KynA.

Mice received daily vehicle or KynA treatment following MI. Animals were euthanized at day 5 post-MI, and peripheral blood were harvested for flow cytometric analysis. n = 6 per group.

A. Flow cytometric plots of monocytes in the peripheral blood of MI mice treated with vehicle or KynA.

B. Percentages and absolute counts of monocytes in the peripheral blood of MI mice treated with vehicle or KynA.

C. Flow cytometric plots of $Ly6C^{hi}$ and $Ly6C^{lo}$ monocytes in the peripheral blood of MI mice treated with vehicle or KynA.

D. Percentages and absolute counts of $Ly6C^{hi}$ and $Ly6C^{lo}$ monocytes in the peripheral blood of MI mice treated with vehicle or KynA.

Statistical comparisons: 2-tailed unpaired t-test was performed in (B) and (D).

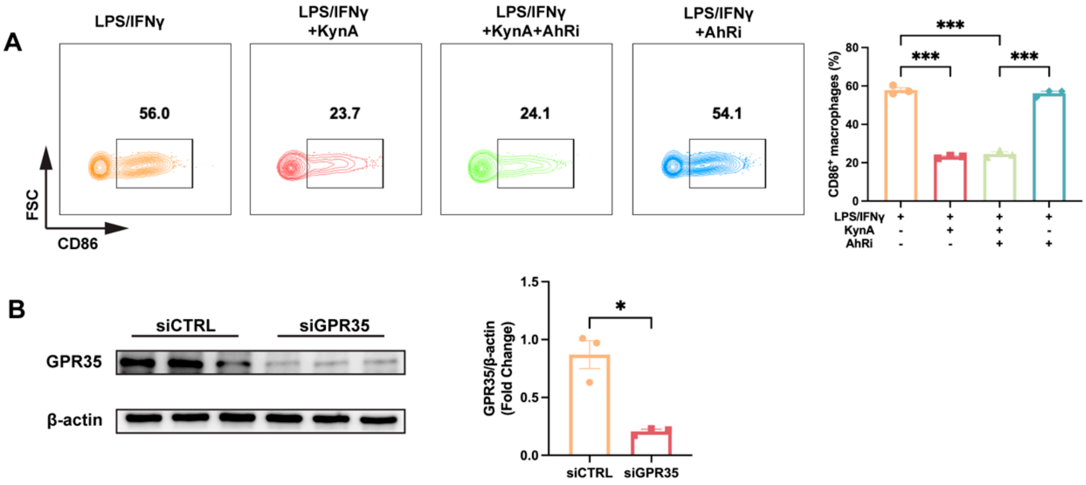

Figure S3. KynA inhibits signaling pathway the proinflammatory phenotype in macrophages through GPR35.

A. Representative flow cytometry plots (**Left**) and percentages (**Right**) of CD86+ macrophages among the indicated groups. n = 3 per group.

B. Confirmation of GPR35 knockdown in BMDMs. Representative Western blotting images (**Left**) and quantification analysis (**Right**) of GPR35 expression in BMDMs treated with control or GPR35 siRNA. n = 3 per group.

Statistical comparisons: two-way ANOVA was performed in (B); 2-tailed unpaired t-test was performed in (B). * $P < 0.05$, *** $P < 0.001$.

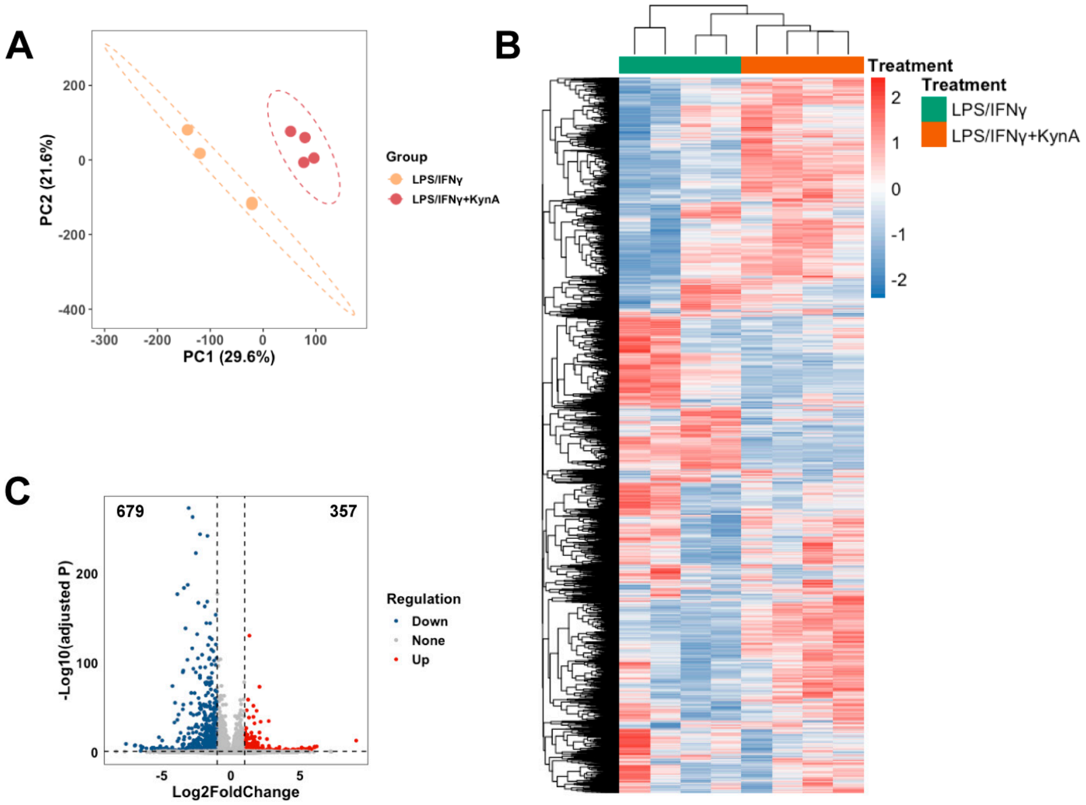

Figure S4. Transcriptomic analysis of cells treated with LPS/IFNγ with or without KynA.

A. Principal component analysis (PCA) of gene expression profiles. n = 4 per group.

B. Heatmap showing the expression levels of DEGs between treatment groups. n = 4 per group.

C. Volcano plot showing differentially expressed genes (DEGs) between LPS/IFNγ and LPS/IFNγ+KynA groups. n = 4 per group.

Supplemental Tables

Supplemental Table S1

Primers used for RT-qPCR and mtDNA measurement

Gene	*Acession*	Primer direction	Sequence
GAPDH	*NM_008084*	F	AGGTCGGTGTGAACGGATTTG
		R	TGTAGACCATGTAGTTGAGGTCA
Il1b	*NM_008361*	F	GAAATGCCACCTTTTGACAGTG
		R	TGGATGCTCTCATCAGGACAG
Tnf	*NM_001278601*	F	CAGGCGGTGCCTATGTCTC
		R	CGATCACCCCGAAGTTCAGTAG
Il6	*NM_031168*	F	CTGCAAGAGACTTCCATCCAG
		R	AGTGGTATAGACAGGTCTGTTGG
Cags	*NM_173386*	F	CAGGAAGGAACCGGACAAGC
		R	CCGACTCCCGTTTCTGCATT
Sting1	*NM_028261*	F	TCGCACGAACTTGGACTACTG
		R	CCAACTGAGGTATATGTCAGCAG
Nos2	*NM_010927*	F	GTTCTCAGCCCAACAATACAAGA
		R	GTGGACGGGTCGATGTCAC
Cox2	-	F	AATCGAGTAGTACTCCCGATTG
		R	TTCTAGGACGATGGGCATGAAA
Atp6	-	F	AATCCAAGCCTACGTTTTCACA
		R	AGTATGAGGAGCGTTATGGAGT
D-loop	-	F	CTATCACCCTATTAACCACTCA
		R	TTCGCCTGTAATATTGAACGTA
Ccl2	*NM_011333*	F	TTAAAAACCTGGATCGGAACCAA
		R	GCATTAGCTTCAGATTTACGGGT
Ccl3	*NM_011337*	F	TTCTCTGTACCATGACACTCTGC
		R	CGTGGAATCTTCCGGCTGTAG
Ccl4	*NM_013652*	F	TTCCTGCTGTTTCTCTTACACCT
		R	CTGTCTGCCTCTTTTGGTCAG
Cxcl10	*NM_021274*	F	CCAAGTGCTGCCGTCATTTTC
		R	GGCTCGCAGGGATGATTTCAA
